# Workforce challenges to implementation of myopia management in Europe: an electronic/telephone expert survey

**DOI:** 10.3389/fopht.2026.1812277

**Published:** 2026-04-22

**Authors:** Annegret Dahlmann-Noor, Line Kessel, Wolf A. Lagrèze, Susana Noval, Jan Roelof Polling, Edoardo Villani, Dominique Brémond-Gignac

**Affiliations:** 1 National Institute for Health and Care Research (NIHR) Moorfields Biomedical Research Centre, London, United Kingdom; 2 Moorfields Eye Hospital National Health Service (NHS) Foundation Trust, London, United Kingdom; 3 University College London Institute of Ophthalmology, London, United Kingdom; 4 Department of Clinical Medicine, University of Copenhagen, Copenhagen, Denmark; 5 Department of Ophthalmology, Copenhagen University Hospital - Rigshospitalet, Glostrup, Denmark; 6 Universitätsklinikum Freiburg, Klinik für Augenheilkunde, Freiburg, Germany; 7 Paediatric Ophthalmology Department, Hospital Universitario La Paz, Madrid, Spain; 8 Universidad Autónoma de Madrid, Madrid, Spain; 9 Erasmus MC, University Medical Center, Rotterdam, Netherlands; 10 Department of Clinical Science and Community Health (DISCCO), University of Milan, Milan, Italy; 11 Eye Clinic San Giuseppe Hospital, Scientific Institute for Research, Hospitalization and Healthcare) (IRCCS) Multimedica, Milan, Italy; 12 University Hospital Necker Enfants malades, APHP, Paris, France; 13 INSERM, UMRS1138, T17, Sorbonne Paris Cití University, Cordeliers Research Center, Paris, France

**Keywords:** adolescent, child, myopia, pathway, public health

## Abstract

The European Medicines Agency and the UK Medicines and Healthcare products Regulatory Agency recently approved the first commercial preparation of low-concentration atropine, Ryjunea, for use in children aged 3 years and older to slow the progression of myopia. Whilst optical approaches have been available for several years, the addition of a pharmacological option will increase the involvement of ophthalmologists in paediatric myopia management. However, little information is available about implementation strategies in different European countries. We conducted an electronic/telephone survey with key opinion leaders in myopia management in Germany, the Netherlands, Denmark, the UK, France, Italy, and Spain. We complemented the survey with figures on population size reported by the World Bank and UNICEF, and workforce numbers reported by the International Council of Ophthalmology, the International Orthoptic Association, and national associations, where available. We collated data in categories reflecting the current provision of eye care for children and workforce availability. Prescribing rights for medicines, spectacles, and contact lenses for children, as well as workforce numbers, including paediatric ophthalmologists, optometrists, and orthoptists, vary significantly across European countries. In general, workforce numbers appear low compared with the number of children and young people in each country. Policies and implementation pathways for myopia management in children are required to ensure equitable access. European guidelines for training in myopia management and national pathways and standards of care should be developed and endorsed by professional organisations to enable children and young people at risk of myopia progression to access appropriate interventions in a timely and economical fashion.

## Introduction

Around the world, myopia (short-sightedness) is an increasing public health challenge, predicted to affect half the world population by 2050, including 740 million children and young people (CYP) (1). Myopia is typically caused by excessive elongation of the eye during childhood and adolescence, driven by CYP spending less time outdoors and more time on near-vision activities, including books and screens (2). Increased eye length is a risk factor for sight-threatening complications in adult life (3). Emerging therapies effectively slow the progression of eye elongation in childhood, such as myopia-control spectacles and contact lenses, and low-concentration atropine eye drops (4), but the implementation of optical and pharmacological approaches and the respective roles of different eye care professionals remain ill-defined (5–7). In particular, the role of workforce availability, access to eye care and healthcare professionals, and reimbursement for interventions are seldom discussed. Across Europe, there is significant variation in healthcare workforce numbers, including ophthalmologists, optometrists, and orthoptists (8), which will impact the implementation and delivery of myopia management (9).

The recent approval of the first commercial formulation of low-concentration atropine to slow myopia progression in children from the age of 3 years by the European Medicines Agency (EMA) (10) and the UK Medicines and Healthcare products Regulatory Agency (MHRA) (11) has intensified the debate about myopia management implementation. Prescribing rights for medicines typically lie with medically qualified practitioners and sometimes orthoptists, but in many countries, the number of these professionals may be small, raising concerns about the roll-out of this safe and well-tolerated medication. In this article, we explore the current landscape of myopia care for children across Europe.

To map the current provision of myopia care for CYP in different European countries, one author (ADN) conducted an electronic/telephone survey with key opinion leaders (KOLs) in myopia management in Germany, the Netherlands, Denmark, the UK, France, Italy, and Spain. Participants were selected based on their publication record in myopia management and prior leadership roles in myopia research. On 27 August 2024, the lead author emailed a list of 18 pre-specified questions to the participants (single contact, no reminders). These covered current prescription practices for medicines and optical treatments (spectacles and contact lenses), reimbursement schemes, workforce numbers, and the duration of waiting times for appointments with eye care providers (**Supplementary Table 1**). Between 28 August 2024 and 10 September 2024, all participants responded. Most provided their replies by e-mail. One participant arranged a video-conferencing call, and the questions were discussed in an interview. On the same day, the participant was provided with a written summary of the interview responses and returned an amended version.

Responses were analysed using a thematic analysis approach (12), with one author familiarising themselves with response contents, colour-coding topics to generate themes, reviewing all responses in light of potential themes and refining these, and finally summarising responses under these themes.

We obtained figures on population size from World Bank reports, on numbers of children from UNICEF reports, and country-level workforce data on numbers of ophthalmologists and orthoptists from databases of the International Council of Ophthalmology and the International Orthoptic Association, respectively; we also checked numbers of optometrists, opticians, and paediatric ophthalmologists with national professional associations, where available.

All invited participants agreed to take part in the survey and gave written consent for anonymised use of demographic and professional data, which are displayed in **Supplementary Table 2**.

## Perspectives: variation in the provision of eye care for children and young people

Four themes were identified in the survey responses: workforce numbers and roles, prescribing qualifications, payment and reimbursement for optical and pharmacological myopia control treatments, and waiting times for appointments to see different eye care professionals.

### Theme 1: Workforce numbers and roles

#### Ophthalmologists

In the countries included, ophthalmologists are employed by the public health sector and work in hospitals. They may also work in community settings or specialist centres (France, Spain, Italy, Denmark, and Germany). The number of paediatric ophthalmologists varies and is often impossible to determine accurately, as there is no register of subspecialty interests (**Table 1**). Defining “paediatric ophthalmologist” is also difficult, as not all countries offer subspecialty training programmes or professional societies for paediatric ophthalmology.

**Table 1:** Calculated estimates of workforce numbers per 10,000 children and young people.

Eye care professionals	Germany	Netherlands	Denmark	United Kingdom	France	Italy	Spain
Ophthalmologists per 10,000 children and young people (calculated, ICO)	5	3	4	2	5	4	4
Ophthalmologists per 10,000 children and young people (calculated, Statistica)	3	2	3	1	4	8	5
Paediatric ophthalmologists per 10,000 children and young people	0.16	0.19	0.13	0.18	0.07	0.44	0.51
Optometrists per 10,000 children and young people	17	4	27	11	2	24	21
Orthoptists per 100,000 children and young people	1.50	1.69	1.5	0.84	1.05	0.89	0.51

There is considerable variation in the eye care workforce for children and young people across countries in Europe. Source population and workforce numbers are shown in **Supplementary Table 3**.

Sources: Population size: World Bank Data Bank (19).

Number of children and young people under age 18 years: UNICEF Data Child Statistics (20).

Number of ophthalmologists: International Council of Ophthalmology (21).

Number of paediatric ophthalmologists: co-authors’ estimates and Royal College of Ophthalmologists 2022 Census (22).

Number of optometrists per 10,000 population: Statistica (23).

Number of orthoptists: Bundesverband Orthoptik Deutschland (24), Nederlandse Vereining van Orthoptisten (25), Dansk Medicinsk Ortoptisk Forening (26), British and Irish Orthoptic Society (17), Syndicat National Autonome des Orthoptistes (27), Associazione Italiana Ortottisti Assistenti in Oftalmologia (28), and UK Health and Care Professions Council (29).

In most countries, general ophthalmologists contribute to children’s eye care, for acute conditions (UK), and/or all paediatric eye conditions they feel competent to manage, such as refractive error and amblyopia management (Germany, Denmark, France, and Spain). Overall, paediatric ophthalmologists seem to be rare: 0.7 to 5 per 100,000 CYP under the age of 18 years, or 1 for every 20,000 to 143,000 CYP (**Table 1**). They mainly provide secondary- and tertiary-care level management, such as treatment for retinopathy of prematurity, surgery for strabismus, childhood glaucoma, and cataract. Established training programmes exist in the UK and France; no formal fellowships are available in the Netherlands, Germany, Denmark, Italy, or Spain.

#### Optometrists

There is great variation in the role of optometrists and opticians across Europe, ranging from established allied health care professional (HCP) status to dispensing optician. In many countries, optometrists working in hospitals play an enhanced role compared with those based in the community.

In the UK, primary eye care, such as sight tests and prescriptions for glasses/contact lenses, is provided by community-based optometrists, many of whom hold advanced qualifications; most UK optometrists can assess children from the age of approximately 5 years, including cycloplegic refraction and prescription of glasses. In other European countries, the role of optometrists and opticians is less well-defined, and training schemes are variable. In Denmark, the Netherlands, and Spain, bachelor’s degree programmes in optometry are available. In Denmark, these can be followed by a master’s degree, but this does not necessarily expand professional roles; optometrists can independently prescribe glasses/contact lenses for children over the age of 10 years, but not diagnose eye conditions. In Germany, training ranges from 3-year apprenticeships to Bachelor of Science and Master’s programmes, but the opportunity for independent practice, including refraction and prescription of glasses, is restricted. In France and Italy, opticians are not recognised as HCPs. In France, they dispense glasses prescribed by ophthalmologists, and in Italy, they can dispense glasses for mild refractive errors without refraction. Whilst optometrists in the UK and the Netherlands can apply cyclopentolate for paediatric refraction, those in France, Spain, Italy, Denmark, and Germany cannot.

#### Orthoptists

Orthoptists are considered healthcare professionals in most countries; they work alongside ophthalmologists and assess children. In France, the Netherlands, and Italy, orthoptists are involved in the management of refractive errors, although in Italy, their role in prescribing glasses for children is controversial. In the UK, whilst orthoptists learn how to carry out refractions, they rarely maintain this skill after qualification. In Denmark, orthoptists are not registered as health care professionals, but many have a prior degree in nursing and are registered as nurses. Working under an ophthalmologist’s supervision, they can carry out refractions and prescriptions of glasses and contact lenses.

### Theme 2: Prescribing qualifications

#### Who can prescribe medicines, including eye drops?

Medically qualified practitioners can prescribe topical eye medicines; this includes ophthalmologists, general practitioners/family doctors, and paediatricians. In practice, topical treatments are overwhelmingly prescribed by ophthalmologists, particularly long-term treatments.

In most countries, optometrists cannot prescribe medicines; an exception is the UK, where approximately 1,000 optometrists hold an advanced qualification to independently prescribe commercially available eye medications.

#### Who can prescribe spectacles and contact lenses for children and young people with simple refractive error?

Ophthalmologists can prescribe spectacles and contact lenses in all European countries, as can optometrists in the UK and opticians in Germany and the Netherlands. In Denmark, children younger than 10 years usually receive their first prescription from an ophthalmologist and often also subsequent prescriptions, particularly if cycloplegia is required. Optometrists can also continue non-cycloplegic refractive care and prescribing for children over the age of 10 years. In Italy, optometrists can dispense glasses for mild refractive errors. A particular issue is cycloplegic refractions, limiting prescribing by optometrists in some countries, such as Spain. In the UK, the Netherlands, Denmark, France (only for adults younger than 50 years), and Italy, orthoptists can also prescribe optical treatments, but practice varies. Prescribing in hospital settings may cause an additional step in the patient journey, compared with countries where community optometrists can refract and dispense.

### Theme 3: Payment and reimbursement for optical and pharmacological myopia control treatments

#### Who pays for children’s glasses and contact lenses?

In most European countries, for simple refractive errors, parents pay for children’s glasses, and reimbursement systems cover part or all of the cost (Germany, Denmark, France, and Spain). In the Netherlands, only lenses for high myopia (over −6.00D) and hypermetropia associated with accommodative esotropia are reimbursed, and in Italy, only those for severe or amblyogenic refractive errors. In the UK, the National Health Service (NHS) pays for children’s glasses with standard single-vision lenses, but not contact lenses. Myopia management glasses are not covered by the NHS, except in Wales; parents have to pay for these.

#### Who pays for low-concentration atropine?

The recent EMA and MHRA approvals of the first commercial preparation of low-concentration atropine will change the prescribing landscape. However, as approvals are for now limited to 0.01% atropine, higher concentrations will continue to be compounded by local hospitals or imported under special license. Prescriptions for compounded formulations can only be issued at selected hospitals and clinics. Funding and reimbursement by public health systems follow local rules: in the Netherlands and France, low-concentration atropine is fully reimbursed; in Spain, partial reimbursement is provided. In Germany, Denmark, the UK, and Italy, parents/carers currently have to cover the cost. In the UK, the National Institute for Health and Care Excellence, the authority that can recommend public reimbursement of medicines and procedures, is carrying out a single technology assessment of low-concentration atropine; this may lead to widespread implementation and adoption (13).

Treatment costs may increase if combination treatment (optical plus pharmacological) is required, which is now often offered to those with fast progression. In addition to treatment costs, families may also incur out-of-pocket expenses for consultation fees, which may or may not be reimbursed; myopia control check-ups routinely take place every 12 months and at shorter, 6-monthly intervals if progression is fast.

### Theme 4: Waiting times for appointments to see different eye care professionals

Waiting times for children’s eye clinic appointments vary considerably between countries and between the types of eye care practitioners. Estimated waiting times for an elective appointment with an ophthalmologist in public health systems tend to be the longest, as many community-based ophthalmologists do not see children, and vary from 2 weeks to 12 months, in some regions even up to 2 years. In Denmark, the UK, France, Italy, and Spain, those covered by private insurance or able to self-pay for their care can attend private clinics, but there may be concerns about the lack of regulation of their practice, with practitioners assessing children without formal paediatric training and qualification other than those during their basic training in ophthalmology. The waiting time to see a community-based orthoptist may be up to 6 months. Community-based opticians and optometrists tend not to have significant waiting times.

## Discussion

This study presents an up-to-date overview of current paediatric eye care, including assessment practices and prescription rights for medicines, spectacles, and contact lenses for children across Europe. It highlights significant variations in competencies and qualifications, and variable ratios of workforce to paediatric population served. Myopia, being a common eye condition, is now affecting nearly 25% of young people in Europe (14) (15), and the advent of new treatments to slow myopia progression in children and young people carries the risk of exacerbating existing waiting times for paediatric eye care appointments. Rising myopia prevalence in childhood will lead to rising prevalence of irreversible sight impairment and blindness, currently predicted to affect 0.84% of the European population by 2075 (16).

Our study is limited by its small participant sample size and potential for selection bias. Myopia progression is a new condition in the lexicon of treatable eye and vision conditions; the number of experts and key opinion leaders is small. The selection of one recognised key opinion leader and paediatric eye care practitioner from each country, therefore, seems appropriate and sufficient to address the questions asked in this study. All participants and co-authors have worked in the new field of myopia control for years and are at the forefront of both research and clinical implementation.

Our findings with regard to workforce challenges in paediatric eye care match those previously described for the UK (9, 17, 18). Whilst we observed that information about workforce levels is not systematically published in all countries and for all types of eye care professionals, the information collated here indicates that many families struggle to access eye care in a timely fashion.

The present study is limited to selected European countries, those with high population numbers. Generalisation to other countries and settings within and outside Europe is not necessarily possible due to differences in healthcare funding and training pathways for eye care professionals.

## Conclusion

The workforce, competency, and regulatory landscape for the prescription of glasses, contact lenses, and eye medication is highly variable across European countries. European guidelines for training in myopia management and national pathways and standards of care should be developed and endorsed by professional organisations to enable children and young people at risk of myopia progression to access appropriate interventions in a timely and economical fashion.

## Data Availability

The raw data supporting the conclusions of this article will be made available by the authors, without undue reservation.

## Supplementary material

Supplementary Table 1 Survey questions.

Supplementary Table 2 Participant characteristics.

Supplementary Table 3 Estimates of workforce numbers.

Supplementary Table 4 Reporting checklist: Consensus-Based Checklist for Reporting of Survey Studies (CROSS) (30).
