# Supplementary material for: Workforce challenges to implementation of myopia management in Europe: an electronic/telephone expert survey
Source: Front Ophthalmol (Lausanne). 2026 Apr 22;6:1812277. doi: 10.3389/fopht.2026.1812277 (PMC13143659; doi:10.3389/fopht.2026.1812277)
Supplement: Supplementary Table 1 — Survey questions. [file DataSheet1.docx]

| Who can prescribe pharmacological agents, such as atropine eyedrops?  Do the prescribers have a financial benefit from prescribing medication, for example a higher re-imbursement tariff?  Are the prescribers based at a hospital, in the community, or both?  Who can prescribe spectacles?  Do the prescribers have a financial benefit from prescribing new glasses, for example a higher re-imbursement tariff?  Are the prescribers based at a hospital, in the community, or both?  Who can prescribe and fit contact lenses?  Do the prescribers have a financial benefit from prescribing contact lenses, for example a higher re-imbursement tariff?  Are the prescribers based at a hospital, in the community, or both?  Who pays for children’s glasses – parents, insurance, public health system?  Who pays for children’s contact lenses – parents, insurance, public health system?  Who pays for children’s low-concentration atropine – parents, insurance, public health system?  How many paediatric ophthalmologists are there in your country (estimate)?  Are general ophthalmologists allowed to assess children?  How many optometrists are there in your country (estimate)?  Are optometrists allowed to use cyclopentolate?  How long do families have to wait for an appointment with an ophthalmologist?  How long do families have to wait for an appointment with an optometrist?? |
| --- |

**Supplementary Table 1. Survey questions.**

| **Age group, n (%)** |  | | |
| --- | --- | --- | --- |
| 40-49 years | 2 (29%) | | |
| ≥50 years | 5 (71%) | | |
| **Sex,** n (%) |  | | |
| Male | 3 (43%) | | |
| Female | 4 (57%) | | |
| **Years since primary qualification**  Mean (Standard Deviation) | 27 (7) | | |
| **Type of professional background**  Ophthalmologist  Orthoptist | 6 (86%)  1 (14%) | | |
| **Type of practice setting, n (%)** |  | | |
| Hospital eye clinic | 4 (57%) | | |
| Hospital eye research facility | 2 (29%) | | |
| Academic eye clinic | 1 (14%) | | |
| **Experience** | 11-100 | 101-500 | ≥500 |
| Number of children and young people managed with any myopia management intervention over the past 5 years n (%) | 1 (14%) | 2 (29%) | 4 (57%) |
|  | ≤5 | 6-10 | 11-20 |
| Number of myopia-related research projects involved in, either as principal or as co-investigator n (%) | 3 (43%) | 2 (29%) | 2 (29%) |
|  | ≤10 | 11-20 | ≥20 |
| Number of publications on a myopia-related topic as author or co-author in the past 10 years. n (%) | 2 (29%) | 3 (43%) | 2 (29%) |
|  | ≤5 | 6-10 | 11-20 |
| Number of myopia-related grant applications led or co-applied for over the past 10 years | 4 (57%) | 2 (29%) | 1 (14%) |

**Supplementary Table 2. Participant characteristics.**

|  | **Germany** | **Netherlands** | **Denmark** | **United Kingdom** | **France** | **Italy** | **Spain** |
| --- | --- | --- | --- | --- | --- | --- | --- |
| **Population** Total population in million (2022) | 83.8 | 17.7 | 5.9 | 67 | 67.8 | 59 | 47.8 |
| Number of children and young people under 18 years of age, in million | 14 | 3.34 | 1.15 | 14.4 | 13.6 | 9 | 7.9 |
| **Ophthalmologists** Number of ophthalmologists per 10,000 population (ICO) | 0.81 | 0.54 | 0.79 | 0.49 | 1.09 | 0.68 | 0.71 |
| Number of ophthalmologists per 10,000 population (Statistica) | 0.49 | 0.38 | 0.5 | 0.22 | 0.88 | 1.26 | 0.89 |
| Calculated total number of ophthalmologists (ICO) | 6788 | 956 | 466 | 3283 | 7390 | 4012 | 3394 |
| Calculated total number of ophthalmologists (Statistica) | 4106 | 673 | 295 | 1474 | 5966 | 7434 | 4254 |
| Number of paediatric ophthalmologists | 230 | around 65 | 15-20 | around 254 | around 100 | around 400 | around 400 |
| **Optometrists**  number of optometrists per 10,000 population (Statistica) | 2.77 | 0.73 | 5.29 | 2.37 | 0.47 | 3.64 | 3.54 |
| Calculated absolute number of optometrists | 23,213 | 1,292 | 3,121 | 15,879 | 3,187 | 21,476 | 16,921 |
| **Orthoptists** absolute number (national associations) | 2,100 | 564 | 18 | 1,212 | 1,430 | around 800 | (around 400, personal communication) |

**Supplementary Table 3. Estimates of workforce numbers.**
Sources: Population size: World Bank Data Bank [19]. Number children and young people under age 18 years: Unicef Data Child Statistics [20].
Number of ophthalmologists: International Council of Ophthalmology [21]

Number of paediatric ophthalmologist: co-authors’ estimates and Royal College of Ophthalmologists 2022 Census [22]

Number of optometrists per 10,000 population: Statistica [23]

Number of orthoptists:, Bundesverband Orthoptik Deutschland [24], Nederlandse Vereining van Orthoptisten [25], Dansk Medicinsk Ortoptisk Forening [26], British and Irish Orthoptic Society [17], Syndicat National Autonome des Orthoptistes [27], Associazione Italiana Ortottisti Assistenti in Oftalmologia [28], UK Health and Care Professions Council [29]

| **Section/topic** | **Item** | **Item description** | **Reported on page #** |
| --- | --- | --- | --- |
| **Title and abstract** | | |  |
| Title and abstract | 1a | State the word “survey” along with a commonly used term in title or abstract to introduce the study’s design. | 1 |
|  | 1b | Provide an informative summary in the abstract, covering background, objectives, methods, findings/results, interpretation/discussion, and conclusions. | 3 |
| **Introduction** | | |  |
| Background | 2 | Provide a background about the rationale of study, what has been previously done, and why this survey is needed. | 4 |
| Purpose/aim | 3 | Identify specific purposes, aims, goals, or objectives of the study. | 4 |
| **Methods** | | |  |
| Study design | 4 | Specify the study design in the methods section with a commonly used term (e.g., cross-sectional or longitudinal). | 4 |
|  | 5a | Describe the questionnaire (e.g., number of sections, number of questions, number and names of instruments used). | 4, Supp Table 1. |
| Data collection methods | 5b | Describe all questionnaire instruments that were used in the survey to measure particular concepts. Report target population, reported validity and reliability information, scoring/classification procedure, and reference links (if any). | 4, Supp Table 1. |
|  | 5c | Provide information on pretesting of the questionnaire, if performed (in the article or in an online supplement). Report the method of pretesting, number of times questionnaire was pre-tested, number and demographics of participants used for pretesting, and the level of similarity of demographics between pre-testing participants and sample population. | n/a |
|  | 5d | Questionnaire if possible, should be fully provided (in the article, or as appendices or as an online supplement). | Supp Table 1 |
| Sample characteristics | 6a | Describe the study population (i.e., background, locations, eligibility criteria for participant inclusion in survey, exclusion criteria). | Page 4, Supp Table 2 |
|  | 6b | Describe the sampling techniques used (e.g., single stage or multistage sampling, simple random sampling, stratified sampling, cluster sampling, convenience sampling). Specify the locations of sample participants whenever clustered sampling was applied. | Page 4 |
|  | 6c | Provide information on sample size, along with details of sample size calculation. | Page 4, Supp Table 2 |
|  | 6d | Describe how representative the sample is of the study population (or target population if possible), particularly for population-based surveys. | n/a |
| Survey  administration | 7a | Provide information on modes of questionnaire administration, including the type and number of contacts, the location where the survey was conducted (e.g., outpatient room or by use of online tools, such as SurveyMonkey). | Page 4 |
|  | 7b | Provide information of survey’s time frame, such as periods of recruitment, exposure, and follow-up days. | Page 4 |
|  | 7c | Provide information on the entry process:  –>For non-web-based surveys, provide approaches to minimize human error in data entry.  –>For web-based surveys, provide approaches to prevent “multiple participation” of participants. | Page 4 |
| Study preparation | 8 | Describe any preparation process before conducting the survey (e.g., interviewers’ training process, advertising the survey). | n/a |
| Ethical considerations | 9a | Provide information on ethical approval for the survey if obtained, including informed consent, institutional review board [IRB] approval, Helsinki declaration, and good clinical practice [GCP] declaration (as appropriate). | Page 4 |
|  | 9b | Provide information about survey anonymity and confidentiality and describe what mechanisms were used to protect unauthorized access. | Page 4 |
| Statistical  analysis | 10a | Describe statistical methods and analytical approach. Report the statistical software that was used for data analysis. | n/a, Page 4 |
|  | 10b | Report any modification of variables used in the analysis, along with reference (if available). | n/a |
|  | 10c | Report details about how missing data was handled. Include rate of missing items, missing data mechanism (i.e., missing completely at random [MCAR], missing at random [MAR] or missing not at random [MNAR]) and methods used to deal with missing data (e.g., multiple imputation). | n/a |
|  | 10d | State how non-response error was addressed. | n/a |
|  | 10e | For longitudinal surveys, state how loss to follow-up was addressed. | n/a |
|  | 10f | Indicate whether any methods such as weighting of items or propensity scores have been used to adjust for non-representativeness of the sample. | n/a |
|  | 10g | Describe any sensitivity analysis conducted. | n/a |
| **Results** | | |  |
| Respondent characteristics | 11a | Report numbers of individuals at each stage of the study. Consider using a flow diagram, if possible. |  |
|  | 11b | Provide reasons for non-participation at each stage, if possible. | Page 4, Suppl Table 2 |
|  | 11c | Report response rate, present the definition of response rate or the formula used to calculate response rate. | Page 4 |
|  | 11d | Provide information to define how unique visitors are determined. Report number of unique visitors along with relevant proportions (e.g., view proportion, participation proportion, completion proportion). | n/a |
| Descriptive  results | 12 | Provide characteristics of study participants, as well as information on potential confounders and assessed outcomes. | Page 4, Suppl Table 2 |
| Main findings | 13a | Give unadjusted estimates and, if applicable, confounder-adjusted estimates along with 95% confidence intervals and p-values. | n/a |
|  | 13b | For multivariable analysis, provide information on the model building process, model fit statistics, and model assumptions (as appropriate). | n/a |
|  | 13c | Provide details about any sensitivity analysis performed. If there are considerable amount of missing data, report sensitivity analyses comparing the results of complete cases with that of the imputed dataset (if possible). | n/a |
| **Discussion** | | |  |
| Limitations | 14 | Discuss the limitations of the study, considering sources of potential biases and imprecisions, such as non-representativeness of sample, study design, important uncontrolled confounders. | Page 6/7 |
| Interpretations | 15 | Give a cautious overall interpretation of results, based on potential biases and imprecisions and suggest areas for future research. | Page 6/7 |
| Generalizability | 16 | Discuss the external validity of the results. | Page 6/7 |
| **Other sections** | | |  |
| Role of funding source | 17 | State whether any funding organization has had any roles in the survey’s design, implementation, and analysis. | Page 7 |
| Conflict of interest | 18 | Declare any potential conflict of interest. | Page 7 |
| Acknowledgements | 19 | Provide names of organizations/persons that are acknowledged along with their contribution to the research. | Page 7 |

**Supplementary Table 4. Reporting checklist: Consensus-Based Checklist for Reporting of Survey Studies (CROSS)** [30].
